# Assessing the Effectiveness of a Solution-Focused Brief Therapy-Based Intervention on Exam Anxiety in First-Year Polish University Students: A Pilot Study with a Randomized Controlled Trial

**DOI:** 10.3390/healthcare13162001

**Published:** 2025-08-14

**Authors:** Natalia Cavour-Więcławek, Aleksandra Różańska, Aleksandra M. Rogowska

**Affiliations:** Institute of Psychology, University of Opole, 45-040 Opole, Poland; natalia@cavour.pl (N.C.-W.); aleksandra.rozanska@uni.opole.pl (A.R.)

**Keywords:** emotional balance, exam anxiety, randomized controlled trial, Solution-Focused Brief Therapy (SFBT), stress, university students

## Abstract

**Background/Objectives**: Exam anxiety is a prevalent issue among university students, affecting both their academic performance and overall subjective well-being. There is an increasing need for efficient psychological interventions to support students. This study aimed to assess the effectiveness of a four-session group intervention based on Solution-Focused Brief Therapy (SFBT) in reducing exam anxiety among 1st-year psychology students in Poland. Additionally, it examined the single-session effects of the SFBT on positive and negative emotions and perceived stress. **Methods**: A pilot study with a randomized controlled trial with a pretest–post-test design was conducted. The 18 participants aged between 19 and 21 years (*M* = 19.22, *SD* = 0.55) were assigned to either the intervention group (SFBT) or a waitlist control group. Exam anxiety was evaluated before and after the intervention. In the experimental group, emotional states and perceived stress were measured before and after each session. **Results**: The two-way analysis of variance ANOVA 2 (therapy: Before, After treatment) × 2 (group: Experimental, Control) showed significant intervention (*p* < 0.05, η^2^_p_ = 0.27) and interaction effects (*p* < 0.05, η^2^_p_ = 0.22). However, the Experimental and Control groups did not differ significantly in exam anxiety (*p* = 0.32, η^2^_p_ = 0.06). Examining changes during each of the four sessions of SFBT in the Experimental group (*n* = 10), the study showed a significant decrease in stress (*p* < 0.05, η^2^_p_ = 0.47) and negative emotion levels (*p* < 0.01, η^2^_p_ = 0.57) while increasing positive emotions (*p* < 0.05, η^2^_p_ = 0.14), comparing emotional states before and after SFBT sessions. **Conclusions**: Even a brief, four-session SFBT intervention is effective in reducing exam anxiety in university students. Moreover, single SFBT sessions were linked to immediate improvements in emotional functioning in the experimental sample. Future research should be repeated to investigate the long-term effects of the SFBT on well-being and include a larger and more heterogeneous sample of university students.

## 1. Introduction

Anxiety is an emotional state characterized by feelings of tension and physiological arousal in response to situations perceived as potentially threatening [1]. While anxiety can play an adaptive role and enhance performance in challenging situations, excessive or chronic anxiety may contribute to the development of various mental illnesses, such as anxiety disorders [2,3,4,5,6]. In a meta-analysis, De Lijster et al. [7] found that the onset of anxiety disorders generally occurs between early adolescence and young adulthood. Research indicates a strong connection between stress and anxiety, emphasizing shared neural pathways [8] and revealing that even mild stress may exacerbate anxiety-related symptoms [9]. According to Cohen [10], perceived stress illustrates how strongly an individual subjectively experiences situations as unpredictable, uncontrollable, or overwhelming. Even minor daily stressors can heighten perceived stress, increasing negative affect and symptoms of anxiety and depression [11,12,13].

Anxiety is a widespread concern among university students, with a reported prevalence of 39% [14,15,16]. Higher levels are particularly noted among female students and those in their early years of study [17]. Mofatteh [18] identified several risk factors contributing to student stress and mental health difficulties, including academic factors such as fear of poor grades, workload demands, and exam pressure. Numerous theoretical models address exam anxiety, also known as test anxiety [19]. Liebert & Morris [20] provided one of the earliest definitions, characterizing it as consisting of two components: a cognitive dimension, which involves concern about performance and the potential consequences of failure, and an emotional dimension, which pertains to somatic arousal in response to perceived stress. Higher levels of exam anxiety among students have been significantly associated with reduced concentration and lower selective attention [21], poorer academic performance [19,22], increased risk of dropout [23], and lower levels of subjective well-being [24,25,26].

According to Diener’s theory [27], subjective well-being consists of cognitive (life satisfaction) and affective (the balance between positive and negative experiences) components. Chattu et al. [28] found that academic performance was significantly associated only with the affective dimension of well-being. Chin et al. [29], in turn, reported that this association was significant exclusively for negative emotions and was mediated by test anxiety. Various interventions have been examined to help students effectively manage and reduce test anxiety. Most studies have focused on evaluating the effectiveness of cognitive-behavioral therapy, study skills training, mindfulness techniques, or exercise-based programs [30,31,32]. While these methods have demonstrated consistent efficacy, a growing body of evidence supports using Solution-Focused Brief Therapy (SFBT) across educational contexts [33,34,35,36].

SFBT was developed in the early 1980s by Steve de Shazer, Insoo Kim Berg, and colleagues at the Brief Family Therapy Center in Milwaukee [37,38,39]. Rather than analyzing problems, the solution-focused approach emphasizes clients’ strengths, resources, and preferred future outcomes where their difficulties are resolved [40]. The therapeutic process prioritizes a collaborative, goal-oriented dialogue, helping clients identify and activate existing strengths, aiming to generate small, purposeful shifts in behavior, language, or perception that can lead to broader change [40,41]. SFBT has evolved to focus more on clients’ detailed descriptions of their preferred future and co-constructing meaning through language rather than emphasizing task-based techniques [42,43]. This shift may be exemplified in the Bruges Model, which not only highlights linguistic sensitivity and contextual awareness but also emphasizes the cognitive dimension of therapy, supporting clients in reshaping habitual meanings through the active presence of the therapist [44]. Findings from a meta-analysis [34] suggest that SFBT demonstrates greater effectiveness in group interventions compared to individual therapeutic settings, with even stronger effects observed in non-clinical populations. Aihie & Igbineweka [45] demonstrated that intervention based on SFBT reduced test anxiety among Nigerian university students throughout eight sessions. In contrast, Kim and Frankin [46], in their review of school-based SFBT interventions, reported that most effective programs involved between four and eight sessions, suggesting that the much shorter formats can still be effective, particularly in educational contexts where time and access are limited.

According to Lahad and Ayalon [47], the BASIC Ph is a meta-model that demonstrates methods for understanding coping and resilience. This model refers to different strategies for coping with anxiety, encompassing six dimensions: beliefs (B), affect (A), social connections (S), imagination (I), cognitive processes (C), and physical activities (Ph). The model is based on the assumptions of Solution-Focused Brief Therapy (SFBT) and integrates various psychological theories and concepts, including Frankl’s Will to Meaning (B), Rogers’ Self-concept Theory (A), Adler’s Holistic Approach (S), De Bono’s lateral thinking (I), Lazarus and Folkman’s cognitive appraisal theory (C), and Pavlov’s psychic reflex concept (Ph). Consequently, this model can serve as a comprehensive framework for implementing diverse psychological interventions and facilitating short-term psychotherapy. The model addresses multiple therapeutic domains, including cognitive skills, emotional regulation, motivation, somatic experiences, and social support systems. This multidimensional approach enables individuals to select the most appropriate coping strategies according to their specific needs. Furthermore, SFBT could demonstrate greater compatibility with individual dispositional factors compared to other therapeutic modalities, such as cognitive-behavioral therapy or mindfulness-based interventions (see Table 1 for a detailed comparison).

Existing research suggests that SFBT enhances positive affect and reduces perceived stress [49,50,51]. However, there is a lack of empirical evidence regarding how each session of SFBT may influence specific psychological states. Reducing exam anxiety can play a key role in academic achievement by increasing academic self-efficacy, which can undoubtedly reduce the risk of dropout and maintain high levels of well-being in students. The present study aims to evaluate the effectiveness of a four-session, group SFBT-based intervention among 1st-year psychology students in Poland. The four-session format was selected to examine whether a shorter SFBT intervention could achieve effects comparable to those reported in longer programs [45], as this would make it more accessible in educational settings, consistent with findings by Kim and Franklin [46]. The primary focus of the intervention was to reduce exam anxiety while also assessing changes in positive/negative emotions and perceived stress through each session. Accordingly, the study tested the following hypotheses:

**H1.**  *Four sessions of SFBT will significantly reduce exam anxiety in the experimental group [34,35,36,45,46,52].*

**H2.**  *The SFBT-based intervention will significantly reduce levels of negative emotions and perceived stress [34,35,50,51].*

**H3.**  *The intervention will significantly increase levels of positive emotions [49,50,51].*

## 2. Materials and Methods

### 2.1. Study Design and Procedure

**Figure 1:**
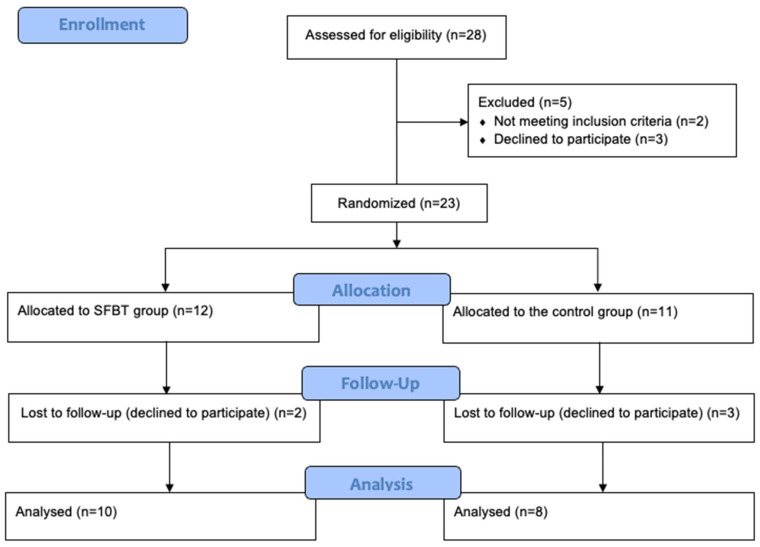
Participant flow diagram.

The hypotheses were examined using a randomized controlled trial (RCT) with a parallel-group design and a 1:1 allocation ratio. The inclusion criterion was enrollment as a 1st-year psychology university student, while the exclusion criterion was prior experience with examinations at the university. To ensure sample homogeneity, only students with no previous academic exam experience were invited to participate so that all group members would face a comparable academic challenge and share similar concerns [53]. Participants were recruited through social media posts, and an in-person invitation was delivered during the lecture. They were informed about the opportunity to participate in a psychological support program offered due to their first university exam session, which was also part of an ongoing research project. They were also notified that some participants would take part in the program immediately, while others would be placed on a waitlist to receive support later. Interested students completed an online eligibility form, which included university email contact information and responses verifying inclusion and exclusion criteria. A total of 28 individuals participated in the qualification process, after which two were excluded due to prior exam experience, and three withdrew before the intervention without providing a reason. The remaining participants (*n* = 23) were randomly assigned to either the experimental group (receiving the SFBT intervention) or a waitlist control group. Random allocation was implemented using a simple alternation method based on the order of registration: individuals with odd numbers were assigned to the experimental group, and those with even numbers were assigned to the control group. A detailed overview of participant flow through the study is presented in the flow diagram (Figure 1). All participants provided written informed consent before participation. The study was approved by the Ethics Committee of Opole University and was preregistered on the Open Science Framework (OSF) prior to data collection. The pre-registration is available at: https://osf.io/qtc9m/ (accessed on 11 August 2025).

### 2.2. Intervention

Participants in the experimental group engaged in a group-based psychological intervention consisting of four weekly sessions based on SFBT, held in January 2025, a month before exams. Each session lasted approximately 60–90 min and was conducted by an SFBT-trained psychologist on university campuses after student classes on Wednesday afternoons. Although the intervention was implemented in a non-clinical context, it was grounded in the core assumptions of SFBT [38,54] and integrated selected elements of the Bruges model proposed by Luc Isebaert [44]. The main objective of the intervention was to alleviate anxiety related to studying, examinations, and post-graduation uncertainty. Specific targets were established on three levels: (1) affective—enhancing positive feelings and diminishing negative emotions; (2) cognitive—reframing academic experiences, broadening perspectives, and identifying personal resources; and (3) behavioral—encouraging more adaptive and goal-oriented actions. The sessions followed a semi-structured format, incorporating each participant’s goals and respecting the principle of client autonomy. Typical SFBT practices [41,55] were employed, including preferred future description, resource identification, exception-seeking, scaling questions, coping questions, relational questioning, complimenting, and co-construction of meaning in context. The structure and content of each session are summarized in Table 2. Three students from the experimental group missed one of the four sessions. However, they attended the remaining meetings and completed all required assessments. Participants in the waitlist control group attended two meetings, limited to completing exam anxiety assessments. They did not receive any form of psychological support during the study period.

### 2.3. Measurements

Standardized self-report instruments were implemented using the traditional paper-and-pencil approach. Participants in both groups completed exam anxiety questionnaires within a pretest–post-test design. In addition, participants in the experimental group completed measures of positive emotions, negative emotions, and perceived stress immediately before and after each of the four sessions.

#### 2.3.1. Exam Anxiety

Given the absence of a Polish version of the self-reported measure of exam anxiety, the currently available self-reported scales are predominantly developed in English and focus on subjective experiences during examinations [56,57]. These scales typically address test anxiety, considered as a state (a temporary response to specific tests) or a trait (a general tendency to experience anxiety in test situations), which can be biased by memory and subjective beliefs. In contrast, our objective was to assess a situational state related to various types of exams (online tests, paper-and-pencil tests, oral exams), which more objectively evaluates physiological and emotional responses to stressful situations, such as exams in an academic context. Regrettably, such a measure is not currently available. Consequently, we sought a recently developed, concise questionnaire to assess the complex physiological, emotional, and behavioral symptoms of anxiety within a specific context, with the intention of adapting it for academic examinations. The following criteria were also applied: it has been utilized in open-access and international studies, translated into multiple languages (including Polish) in various populations (including university students), and it has demonstrated excellent psychometric properties, as well as high reliability and validity. The Fear of COVID-19 Scale (FCV-19S) met all the required criteria [58]. FCV-19S was translated and validated in more than 20 languages worldwide, showing a stable unidimensional structure, excellent reliability, and robust psychometric properties [59].

Exam anxiety was measured by a modified Polish version of the Fear of COVID-19 Scale [60,61]. The original instrument demonstrates satisfactory psychometric properties and captures dimensions that correspond to components of test anxiety [20]. The adaptation involved replacing selected terms in the original version: “COVID-19” was replaced with “exams,” “death from coronavirus” with “consequences of failing exams,” “worrying about getting coronavirus” with “worrying about upcoming exams,” and “watching news about COVID-19” with “hearing news or discussions about exams. The scale includes seven items and is rated on a five-point Likert scale ranging from “I disagree” to “I strongly agree.” A higher score on the scale indicates a greater level of exam anxiety. We conducted preliminary validation of this modified questionnaire in a sample of 86 university students, which indicates a one-factor structure and high reliability assessed by Cronbach’s α = 0.90, and also good criterion validity (correlation with generalized anxiety disorder symptoms with Spearman’s Rho = 0.79, *p* < 0.001). A detailed description of the validation, along with the entire questionnaire, is included in the Supplementary Materials. The internal consistency of the scale was α = 0.81 at pretest and α = 0.77 at post-test.

#### 2.3.2. Stress

Perceived stress was assessed using a single-item Visual Analogue Scale (VAS) ranging from 0 (“no stress at all”) to 10 (“extreme stress”) [62]. Participants marked their current level of stress on a horizontal line. Although the VAS consists of only one item, it is a widely used and psychometrically supported tool for assessing subjective stress. Lesage and Berjot [63] reported a strong correlation with the Perceived Stress scale (PSS; *r* = 0.68), a finding that was further supported by similar results in subsequent studies by Lesage et al. [64] and Dutheil et al. [65]. The use of the VAS in stress assessment has also demonstrated utility in academic contexts [66]. In addition to stress, the VAS has also been applied to the assessment of anxiety-related symptoms [67,68]. Given the repeated-measures design of the present study, in which stress levels were assessed multiple times across the intervention, the VAS was particularly well-suited as a brief and validated tool. Unlike commonly used multi-item stress questionnaires, which typically assess perceptions of stress over a more extended period (e.g., the past week or month), the VAS enables the capture of immediate, momentary affective states. Its use, therefore, is aligned with the study’s objective to track short-term, session-to-session changes in subjective stress with minimal response burden. The reliability of the VAS was examined using intraclass correlation (ICC) [69]. The reliability of the VAS in the current study was good ICC3, k = 0.629 (95% CI = 0.023, 0.896), considering 10 raters and four measurements [70].

#### 2.3.3. Positive and Negative Emotions

The Scale of Positive and Negative Experience developed by Diener et al. [71] in the Polish adaptation by Kaczmarek and Baran [72] was used. The assessment consists of 12 items, divided into two subscales: positive experiences (SPANE-P) and negative experiences (SPANE-N). Participants indicated the intensity of their momentary feelings using a five-point Likert scale ranging from 1 (not at all) to 5 (very strongly). In addition, the Affect Balance (SPANE-B) score can be computed by subtracting the SPANE-N from the SPANE-P, yielding a total score ranging from −24 to +24. Higher SPANE-B scores reflect a more positive emotional balance. Internal consistency was assessed for each session separately. For the SPANE-P, Cronbach’s alpha coefficients before sessions 1–4 were 0.94, 0.92, 0.87, and 0.94, respectively, and after the sessions, 0.91, 0.94, 0.90, and 0.95. The SPANE-N alpha values before the sessions were 0.87, 0.87, 0.82, and 0.81, while post-session values were 0.76, 0.81, 0.89, and 0.86. Due to the small sample size and the momentary nature of the measurement, some SPANE-N items were excluded from the reliability analysis because they showed no variance across participants. Items 8 (sad) and 11 (angry) were removed from the first post-session and third pre-session measurements; items 6 (unpleasant) and 11 from the third pre-session measurement; and items 9 (afraid) and 11 from the fourth pre- and post-session. Despite these exclusions, internal consistency estimates remained acceptable.

### 2.4. Participants’ Characteristics

The required sample size for the exam anxiety analysis was calculated using G*Power. A priori power analysis showed a minimum *N* = 34 participants to be expected for a repeated-measures ANOVA with a between–within interaction (2 groups × 2-time points), ƒ = 0.25, α = 0.05, and power = 0.80. For the repeated-measures analyses involving positive and negative affect and perceived stress (eight time points, within factors), the required sample size was *N* = 23. Participation was voluntary, and the sample was recruited based on accessibility and willingness to participate in a psychological support program. The final sample consisted of 18 students (*n* = 17 women) aged between 19 and 21 years (*M* = 19.22, *SD* = 0.55). All students were in their 1st year of psychology studies at the same university. They all attended the same lectures and belonged to three exercise groups in which the same lecturers presented a standard study curriculum.

After allocation to experimental and control groups, we preliminarily screened all students across disorder symptoms. Depression symptoms were examined using the nine-item Patient Health Questionnaire (PHQ-9) [73,74], general anxiety disorder using the seven-item General Anxiety Disorder scale (GAD-7) [75] in Polish translation, and insomnia symptoms using the eight-item Athens Insomnia Scale (AIS-8) [76] based on the Polish validation by Fornal-Pawłowska et al. [77]. We performed a preliminary analysis to check the assumptions for parametric tests. The Shapiro-Wilk test showed normal distribution for depression symptoms (S-W = 0.96, *p* = 0.667), generalized anxiety symptoms (S-W = 0.92, *p* = 0.148), and insomnia symptoms (S-W = 0.94, *p* = 0.234), but non-normal distribution for age (S-W = 0.60, *p* < 0.001). Test of equality of variances (Levene’s) showed homogeneity for all variables of interest, including age [*F*(1, 16) = 2.12, *p* = 0.165], depression symptoms [*F*(1, 16) = 0.09, *p* = 0.764], generalized anxiety symptoms [*F*(1, 16) = 3.19, *p* = 0.093], and insomnia symptoms [*F*(1, 16) = 0.02, *p* = 0.904]. The results of Student’s *t*-test showed that the experimental group does not differ from the control group in age, symptoms of depression, anxiety, and insomnia (Table 3). The sample size in the present study aligns with previous pilot research on solution-focused group interventions. Notably, Zhang et al. [78] implemented a 5-week SFBT group intervention with 18 university students, and Pu et al. [79] demonstrated meaningful outcomes in a sample of 26 participants. In addition, we conducted post-hoc power calculations based on observed effect sizes, indicating that the collected data were sufficient. Detailed post-hoc power estimates are presented in the results section.

### 2.5. Statistical Analysis

The parametric properties of the data were tested using mean (*M*), standard deviation (*SD*), skewness, kurtosis, and the Shapiro-Wilk normality test. Missing data were addressed through the application of multiple imputation by chained equations (MICE), with 10 iterations in the total conditional specification. The methods employed for imputing missing data included the missing at random (MAR) approach and linear regression imputation (LRI). Multiple linear regression imputation is one of the best methods to deal with missing data, in which missing values are replaced by regression using other variables as parameters. In this study, each variable with missing data was conditionally imputed based on a linear regression model, utilizing the same variable from other measurements derived from one of four experimental sessions. For instance, if a stress value was missing before the third session for a particular individual, the stress values recorded before sessions 1, 2, and 4 were used as predictors to impute the missing data for session 3.

All variables (including exam anxiety, stress, positive emotions, and negative emotions) met the criteria, and we carried out parametric tests on the following tuples. To examine the effect of SFBT on exam anxiety, we used a two-way repeated measures analysis of variance (ANOVA), with main between-effect for two groups (Experimental, Control), one within-effect for two measurements (Before intervention, After intervention), and interaction effect between group and intervention (between and within). To test changes in stress, positive and negative emotions, and emotional balance across the four SFBT-based sessions, we used a two-way repeated measures ANOVA with a day of intervention as one factor (SFBT day) and repeated measures before and after each session as the other factor (Retest). Partial eta-squared was used to assess effect size, and the Bonferroni post-hoc test was used to examine pairwise comparisons. All statistical analyses were performed using JASP version 0.19.3.0 for Windows. The post-hoc method was also used to test the strength of our findings using G*Power software ver. 3.1.

## 3. Results

### 3.1. Effect of SFBT Intervention on Exam Anxiety

**Figure 2:**
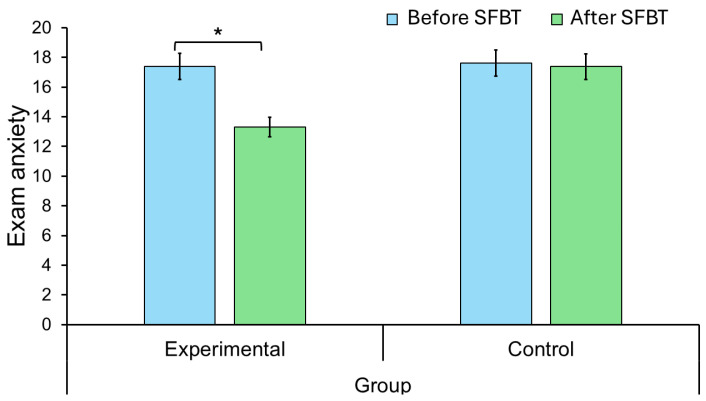
Changes in fear of examination as a function of group and Solution-Focused Brief Therapy (SFBT). Error bars are 95% confidence intervals. * *p* < 0.05.

The two-way ANOVA 2 (time: Before, After treatment) × 2 (group: Experimental, Control) was performed to examine the effect of SFBT on exam anxiety among students (Table 4, Figure 2). The assumptions were met for the ANOVA test, including normality (Shapiro-Wilk test was S-W = 0.96, *p* = 0.605) and homogeneity of variance (Levene’s test *F*(1, 16) = 0.001, *p* = 0.973) in exam anxiety. The effect of the SFBT intervention on exam anxiety was significant (*p* < 0.05, η^2^_p_ = 0.27). The Bonferroni post-hoc test showed exam anxiety scores were significantly reduced after four SFBT-based sessions, compared to measurement before treatment was started, Δ*M* = 2.18, *SE* = 0.90, *t*(16) = 2.41, *p* = 0.029, Cohen’s *d* = 0.45. Groups did not differ in exam anxiety (*p* = 0.32, η^2^_p_ = 0.06). However, the effect of interaction between therapy (Before SFBT, After SFBT) and group (Experimental, Control) was significant (*p* < 0.05, η^2^_p_ = 0.22). The Bonferroni post-hoc test demonstrated that the experimental group reduced exam anxiety during SFBT, Δ*M* = 4.10, *SE* = 0.1.21, *t*(16) = 3.40, *p* = 0.022, Cohen’s *d* = 0.86.

### 3.2. Changes in Self-Reported Stress Levels During SFBT Intervention

**Figure 3:**
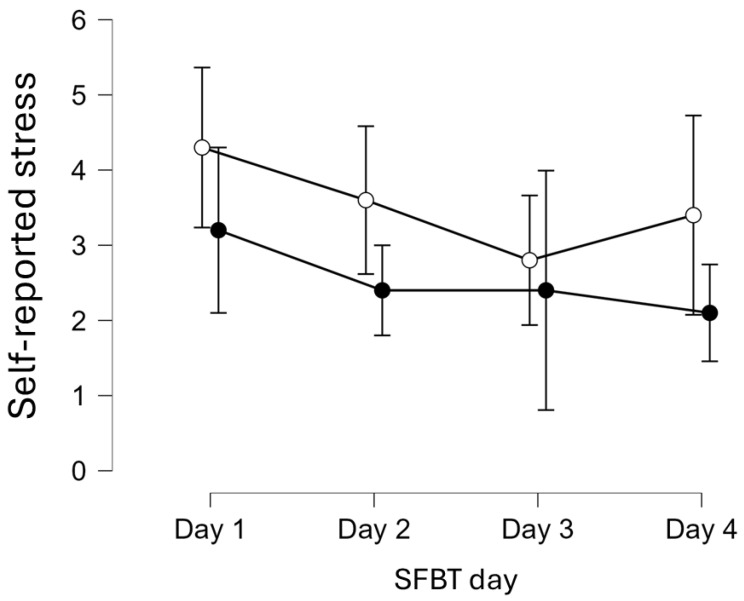
Changes in self-reported stress levels across four days of Solution-Focused Brief Therapy (SFBT) in two measurements. Note. White circle = before SFBT, black circle = after SFBT, error bars are 95% confidence interval.

Changes in stress levels were examined using two-way repeated measures ANOVA in the experimental group (*n* = 10). The assumptions were met for the ANOVA test, including normality (Shapiro-Wilk test was S-W = 0.92, *p* = 0.322) and sphericity (Mauchly’s W = 0.43, χ^2^(5) = 6.53, *p* = 0.262) in perceived stress. Stress levels did not change significantly during the following 4 days of SFBT since the effect for SFBT day was insignificant (*p* = 0.26, η^2^_p_ = 0.14). The effect of retesting during each day of SFBT was significant (*p* < 0.05, η^2^_p_ = 0.47). The Bonferroni post-hoc test showed that stress levels decreased compared to pre-SFBT and post-SFBT states, with a moderate effect size, Δ*M* = 1.00, *SE* = 0.36, *t*(9) = 2.80, *p* = 0.021, Cohen’s *d* = 0.62. However, the interaction between SFBT day and retest (before and after) during each SFBT session was insignificant (*p* = 0.32, η^2^_p_ = 0.12). Detailed results are presented in Table 5 and Figure 3.

### 3.3. Changes in Positive and Negative Emotions Across Four Sessions of SFBT Intervention

**Figure 4:**
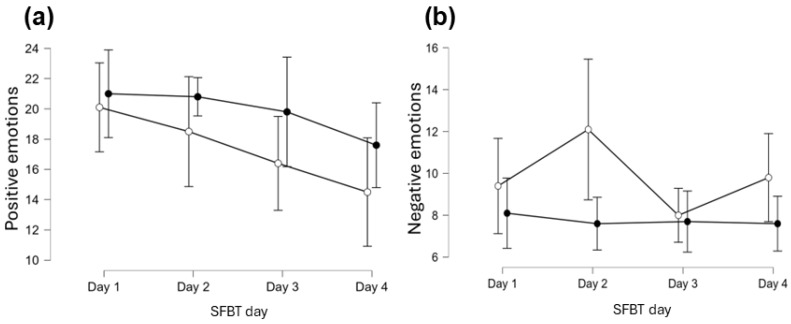
Changes in (**a**) positive emotions, and (**b**) negative emotions across 4 days of Solution-Focused Brief Therapy (SFBT) in two measurements. Note. White circle = before SFBT, black circle = after SFBT, error bars are 95% confidence interval.

The two-way repeated measures ANOVA were performed to examine changes in emotional states during experimental sessions (*n* = 10). The assumptions were met for the ANOVA test, including normality (Shapiro-Wilk test was S-W = 0.87, *p* = 0.090) and sphericity (Mauchly’s W = 0.31, χ^2^(5) = 9.04, *p* = 0.110) in positive emotions. Results are presented in Table 6 and Figure 4. Although levels of positive emotions did not change significantly across the four SFBT days (*p* = 0.10, η^2^_p_ = 0.20), they increased significantly within each session (*p* < 0.05, η^2^_p_ = 0.14), as indicated by comparisons of states before and after each session (*p* < 0.05, η^2^_p_ = 0.14) when compared to states before and after the intervention, Δ*M* = −2.43, *SE* = 0.85, *t*(9) = −2.87, *p* = 0.02, Cohen’s *d* = −0.44. There was no interaction effect between Day and Retest in positive emotions (*p* = 0.23, η^2^_p_ = 0.15).

The assumptions were met for the ANOVA test, including normality (Shapiro-Wilk test was S-W = 0.86, *p* = 0.073) and sphericity (Mauchly’s W = 0.51, χ^2^(5) = 5.27, *p* = 0.388) in negative emotions. The overall level of negative emotions did not change significantly in the following days of intervention (*p* = 0.33, η^2^_p_ = 0.12). However, during SFBT sessions (*p* < 0.01, η^2^_p_ = 0.57), negative emotions decreased significantly, Δ*M* = 2.08, *SE* = 0.60, *t*(9) = 3.48, *p* = 0.007, Cohen’s *d* = 0.63. In addition, an interaction effect between Day and Retest was also significant for negative emotions (*p* < 0.05, η^2^_p_ = 0.32).

### 3.4. Post-Hoc Power Analysis for the Current Study Sample

To ensure that the results of this study had adequate statistical power, we conducted a post-hoc power analysis using the effects obtained in the studies and using G*Power software. The power for effect of SFBT on exam anxiety (2-way ANOVA) was 0.99, considering the reported values for the effect size (ƒ = 0.61), significant level (α = 0.05), total sample size (*N* = 18), two groups (Experimental, Control), and two measurements (Before SFBT, After SFBT), and also a correlation between repeated measures (*r* = 0.60). We calculated the post-hoc power analysis for changes in stress level during four sessions of SFBT, examined by using two-way repeated measures ANOVA in the experimental group only. The post-hoc power of the findings was 1.00, taking into account the reported effect sizes (ƒ = 0.92), significance level (α = 0.5), total sample size (*N* = 10), and eight measurements (4 days × 2 measurements: before and after SFBT), as well as the correlation between repeated measures (*r* = 0.21). Taking into account the changes in the level of positive emotions, post-hoc power analysis showed value of 1.00, considering the reported effect size (ƒ = 0.96), significance level (α = 0.5), total sample size (N = 10), and eight measurements (4 days × measurement before and after SFBT), as well as the average correlation between repeated measures (*r* = 0.21). Similarly, the result of post-hoc power analysis showed a value of 1.00 for changes in negative emotions across the four SFBT sessions, considering the current effect size (ƒ = 1.15), significance level (α = 0.5), total sample size (*N* = 10), and eight measurements (4 days × measurement before and after SFBT), as well as the correlation between repeated measures (*r* = 0.43).

## 4. Discussion

The high prevalence of anxiety among university students represents a significant concern [14,15,16]. Lifestyle changes, relocation, financial constraints, and academic demands all contribute to an increased risk of developing mental health problems [18]. A particularly critical moment for many students is their first formal evaluation through university examinations, often accompanied by heightened levels of exam anxiety [17]. The present study aimed to examine whether a Solution-Focused Brief Therapy-based intervention could effectively support students during this specific period.

The focus and aims differ between SFBT and CBT (see Table 1). The results of this study show that SFBT can reduce pre-exam anxiety and enhance resilience. According to Csirmaz et al. [80], CBT reduced test anxiety between pre- and post-intervention. Their treatment group participated in an 8-week program that included relaxation, skill training, and cognitive-behavioral methods. Our program consisted of only four sessions, yet the results confirm its effectiveness. The results of the study partially supported all three hypotheses. A significant reduction in exam anxiety was observed in the experimental group, compared to the control group, confirming the effectiveness of the group SFBT-based intervention. Effect size for ANOVA was significant (*p* < 0.05) and large for treatment and interaction between treatment and group but was insignificant (*p* > 0.05) and medium for group effect. This may suggest that short-term SFBT does indeed provide only short-term effects. It would probably be necessary to compare four sessions with an 8-week cycle in the future.

This finding is consistent with previous research supporting the use of SFBT in education [34,35,36,46] and reinforces the conclusions drawn by Aihie and Igbineweka [45] regarding its efficacy in reducing exam anxiety. The current study extends these findings by demonstrating the intervention’s effectiveness in a Polish cultural context and within only four sessions [46]. The effect may be attributed to the future-oriented and resource-based nature of SFBT, which supports students in reframing stressful academic situations and identifying coping mechanisms. Moreover, engaging in a group context allows individuals to gain insight from the narratives of others, which may be particularly useful in broadening one’s perspective [52]. It is noteworthy that the baseline mean level of anxiety for the total sample was *M*_GAD-7_ = 5.34, with no significant difference observed between the control group and the experimental sample. Generally, GAD-7 scores ranging from 5 to 9 indicate mild anxiety symptoms, which are noticeable but typically manageable. Two students who exhibited a risk of depression and anxiety disorder were excluded from the statistical analyses, ensuring that the student sample was homogeneous in terms of anxiety symptoms.

The repeated-measures analysis revealed significant post-session reductions in negative emotions and perceived stress, along with improvements in positive emotions. Unfortunately, the effects did not last in the following days of the session, and no continuous increase in students’ well-being was observed. Also, the groups did not differ significantly from each other, suggesting that the treatment works for a short time, and only immediately after the session did the students feel better. Furthermore, the effect size of SFBT on stress level after each session was large and significant (*p* < 0.05). The effect size for the subsequent 4 days of therapy and the interaction effect size were medium but insignificant (*p* > 0.05). Taking into account SFBT sessions on positive emotions, only repeated measurement during a particular day of treatment was significant with a large effect size, while day of treatment and interaction effects remained insignificant but with a medium effect size. In contrast, SFBT significantly reduced negative emotions in students, with a large effect size, during singular treatment sessions, as well as in the interaction between group and treatment.

These findings align with previous research indicating that SFBT fosters hope and optimism while reducing levels of distress [49,50,51]. All effects were statistically significant and characterized by large effect sizes, suggesting that even a single SFBT session may produce short-term emotional benefits. Kim and Franklin [81] emphasize that SFBT actively promotes the experience of positive emotions through its interventions. The intervention integrated selected elements of the Bruges Model [44], which highlights the value of cultivating emotions such as joy, gratitude, and acceptance, which may have contributed to amplifying the observed benefits. However, no sustained effects were observed in improving overall emotional well-being or reducing stress over time, which contrasts with previous findings [34,49]. It is important to note that the measurements of positive and negative emotions referred to the immediate emotional state at the time of assessment (i.e., before and after each session). Therefore, the lack of long-term change may be considered a realistic outcome. It is unlikely that a few sessions focused primarily on exam anxiety would produce enduring changes in broader emotional state indicators. The timing of the measurements, conducted shortly before the examination period, was likely associated with heightened stress levels. As demonstrated by Pascual-Leone [82], emotional change trajectories during psychological interventions often follow non-linear patterns. It is also worth noting that the persistence of negative emotions across sessions may be partially attributed to stable personality traits such as neuroticism, which is strongly associated with a heightened tendency to experience negative effects. Since neuroticism was not controlled for in the current study, future research should consider including it as a covariate to better understand individual differences in emotional responses to brief interventions.

It should be noted that the sample selection was guided not only by practical considerations and the pilot nature of the study, but also by theoretical premises relevant to group-based interventions. One such premise was the deliberate inclusion of a homogeneous group in terms of academic experience. According to Yalom’s group therapy factors, “universality” refers to the recognition of shared experiences and emotional responses among group members, which can foster a sense of connection and mutual understanding [53]. In the present study, all participants were 1st-year psychology students who had not yet taken part in any academic exams. They were all facing a comparable academic context—limited time to study large volumes of material, a high number of assessments, and demanding course requirements. By ensuring similarity in academic experience, we aimed to create a group setting in which participants could more easily relate to one another and engage in meaningful, solution-focused dialogue. While individual differences such as personality traits remained uncontrolled, reducing variability in situational factors allowed us to focus the intervention on managing exam anxiety in response to a shared academic challenge.

The post-hoc power analysis indicates clearly that the sample size for a study was appropriate for the statistical tests and effects found in the data, with 99–100% of confidence in the obtained results. Overall, the post-hoc power analysis ensures the present study has a high enough probability (power) of detecting a real effect while minimizing the risk of false negatives (Type II errors). Likely, a larger sample size could unnecessarily waste the time and effort of researchers and participants as well. Studies with unnecessarily large sample sizes can be expensive and may detect very small, trivial effects that are not meaningful in real-world contexts. Since there have been no similar studies before, we were forced to calculate the minimum number of participants, assuming the expected average effects of statistical tests. It should be noted that power analysis estimates the probability that a study will correctly reject a false null hypothesis (meaning it will find a real effect if one exists). Setting appropriate levels for alpha (significance level) and power helps researchers minimize both the chance of incorrectly rejecting a true null hypothesis (Type I error) and the chance of failing to detect a real effect (Type II error). Our findings indicate that large effects were achieved within four SFBT sessions, and, therefore, the current sample size was more than adequate. By ensuring adequate power, the study can increase confidence that the results of our research are accurate and replicable.

### Study Limitations and Future Research Directions

This study had certain limitations. First, due to the absence of follow-up assessments, it remains unclear whether the observed effects were sustained over time. Second, the study did not examine its direct impact on students’ academic performance. Third, all measures relied on self-reported data. In addition, at several measurement points, certain SPANE-N items had to be excluded from the reliability analysis due to zero variance, which may have influenced the stability of those subscale scores. In this study, we used a modified scale to measure exam anxiety, as well as a single-item stress measure. Although the reliability of these scales was good, future studies should use other validated and specific questionnaires for these variables, psychophysiological methods, as well as qualitative methods that could deepen the obtained results. Longitudinal follow-up would also be advisable to examine the duration of the effect of SFBT. Another limitation is that the study included only students without prior experience in exams. While this ensured sample homogeneity, it also narrows the generalizability of the findings. Future studies should consider including students with more diverse academic backgrounds, such as those from different fields of study and with varying levels of exam-related experience, to better understand for whom SFBT interventions are most effective. Finally, the relatively small sample size limits the generalizability of the findings. However, the large effect sizes observed suggest that the intervention had a meaningful impact on this specific group.

Alternative explanations for research findings, such as expectancy effects, the Hawthorne effect, and social desirability bias, can substantially influence the validity of results. For instance, it is conceivable that participants deduced expectations of enhanced well-being (characterized by reduced levels of stress, anxiety, and negative emotions, and increased levels of positive emotions following each therapy session) from the research procedure. Additionally, some participants may have exhibited a propensity to alter their behavior upon realizing they were being observed or studied. This Hawthorne effect can result in improvements in performance or behavior that are neither sustainable nor generalizable to non-experimental contexts. Social desirability bias refers to the inclination of individuals to respond to questions in a manner they perceive as socially acceptable or likely to be viewed favorably by others, rather than providing truthful responses. This bias can lead to inaccurate data and distort the outcomes of studies that rely on self-report measures. These biases can prompt participants to modify their behavior or responses, complicating the determination of whether observed changes are attributable to the experimental manipulation or these confounding factors. Although the inclusion of a control group may have somewhat mitigated these effects, they need to be better controlled in the future to limit their influence on the results of further studies.

Despite its limitations, the study findings are worth exploring. Further research should aim to replicate these findings in larger and more diverse samples and include follow-up assessments to evaluate the durability of effects and to examine the potential impact of SFBT on academic performance. The study also holds important practical implications. The results suggest that even a brief, four-session SFBT intervention may effectively support students. Given its demonstrated efficacy, brief duration, and low cost, SFBT appears to be an attractive form of psychological support within university counseling services.

## 5. Conclusions

The present pilot study provides evidence that an SFBT-based intervention may serve as an effective and accessible form of psychological support for university students facing various academic challenges. Notably, the findings suggest that even a single session of SFBT can enhance emotional functioning. However, due to the small group, these pilot study results should be treated with caution. Future research should include more extended follow-up periods, more diverse samples, and assessments of academic outcomes.

## Figures and Tables

**Table 1:** Comparison of SFBT and CBT: theoretical framework analysis based on Jordan et al. [48].

Elements of Comparison	Solution-Focused Brief Therapy	Cognitive Behavioral Therapy
Focus	Solution building; positive topics: strength and resources	Problem solving; negative topics: problems and situational difficulties
Kind of therapy	Solution-building model; Bruges model	Behavior therapy; cognitive therapy; dialectial behavior therapy; rational emotive behavior therapy; mindfulness-based cognitive therapy
Aim	Generating ideas to do something better in the future	Problem-solving approach; identify problems; generate the best response to fit a situation
Language	Tool for co-constructing realities	Transmitting information back and forth
Scaling question	How good is the client’s ability	How bad an experience was, or how bad the level of the problem that the client is experiencing
View of clients	Resources of clients, and it is not necessary to identify deficiencies and pathologies	Unhealthy and faulty cognitions—problematic behaviors
Therapists	Take a “not-knowing” stance, they are asking questions to highlight client resources	Expert position, helping to change faulty thinking
Aspects and techniques	Cognitions and behaviours; based on presenting topic by client; homework; scaling question and goal setting	Cognitions and behaviours; based on presenting topic by client; homework; scaling question and goal setting

**Table 2:** Session-by-session content.

Session	Key Activities	SFBT Practices
First, Week 1	Introduction and group rules, discussion about studying and exam session, identification of concerns, resources, and expectations, setting individual goals, observational task: “What helps you feel better?”	Goal setting, resource identification, exception-seeking, future orientation, normalizing
Second, Week 2	Change check using the EARS technique, “Three Questions for a Good Life,” group discussion of an observational task, and planning useful/different actions	EARS (Elicit, Acknowledge, Reinforce, Summarize), positive affect elicitation, compliments, relational use of the group as a resource
Third, Week 3	Change check using the EARS technique, “Three Questions for a Good Life,” readiness for exams, 54,321 grounding technique with Acceptance/Joy/Gratitude.	EARS, positive affect elicitation, scaling questions, coping questions, focused on strengths
Fourth, Week 4	Change check using the EARS technique, “Three Questions for a Good Life,” “Letter from the future” exercise (vision of life post-graduation), identification of strengths and self-complimenting, continuity discussion: “What do you want to remain unchanged?”, final feedback and summary	EARS, positive affect elicitation, preferred future visualization, self-complementing, principle of continuity, feedback

**Table 3:** Student’s *t*-test comparing age, depression, anxiety, and insomnia symptoms between the control and experimental groups.

Variable	Control (*n* = 8)		Experimental (*n* = 10)		*t*(16)	*p*	*d*
	*M*	*SD*	*M*	*SD*			
Age	19.13	0.35	19.30	0.68	36.50 #	0.681	−0.314
Depression symptoms	8.25	3.41	6.80	3.08	0.95	0.358	0.449
Generalized anxiety symptoms	4.88	2.80	5.80	4.85	−0.48	0.639	−0.227
Insomnia symptoms	5.75	3.33	6.80	3.43	−0.65	0.522	−0.310

Note. # Mann-Whitney *U*-test.

**Table 4:** Two-way analysis of variance (ANOVA) statistics for exam anxiety.

	Experimental (*n* = 10)		Control (*n* = 8)					
Variable	*M*	*SD*	*M*	*SD*	Effect	*F*(1, 16)	*p*	η^2^_p_
SFBT					T	5.79	0.029	0.266
Before	17.40	5.36	17.63	4.87	G	1.06	0.318	0.062
After	13.30	4.72	17.38	4.00	T × G	4.54	0.049	0.221

Note. SFBT = Solution-Focused Brief Therapy, T = therapy, G = Group, T × G = interaction between therapy and group. *N* = 18.

**Table 5:** Changes in mean levels of self-reported stress before and after Solution-Focused Brief Therapy during the subsequent 4 days.

SFBT Day	Before SFBT		After SFBT		Effect	*F*	*df*	*p*	η^2^_p_
	*M*	*SD*	*M*	*SD*					
1	4.30	1.57	3.20	1.14	D	1.43	3, 27	0.257	0.137
2	3.60	1.84	2.40	1.27	R	7.83	1, 9	0.021	0.465
3	2.80	1.14	2.40	2.01	D × R	1.22	3, 27	0.323	0.119
4	3.40	2.32	2.10	1.29					

Note. SFBT = Solution-Focused Brief Therapy, D = day of the SFBT (1, 2, 3, 4), R = retest during a given SFBT session (before SFBT, after SFBT), D × R = interaction between day of SFBT and retest during the SFBT day. *N* = 10.

**Table 6:** Changes in mean levels of positive emotions, negative emotions, and emotional balance before and after Solution-Focused Brief Therapy intervention during the subsequent 4 days.

	SFBT Day	Before		After						
Variable		*M*	*SD*	*M*	*SD*	Effect	*F*	*df*	*p*	η^2^_p_
Positive emotions	1	20.10	4.89	21.00	5.19	D	2.28	3, 27	0.102	0.202
	2	18.50	5.60	20.80	4.42	R	8.22	1, 9	0.019	0.477
	3	16.40	4.97	19.80	5.37	D × R	1.54	3, 27	0.226	0.146
	4	14.50	6.43	17.60	6.54					
Negative emotions	1	9.40	4.03	8.10	3.07	D	1.19	3, 27	0.332	0.117
	2	12.10	6.17	7.60	2.01	R	12.11	1, 9	0.007	0.574
	3	8.00	2.06	7.70	2.06	D × R	4.13	3, 27	0.016	0.315
	4	9.80	2.90	7.60	1.90					

Note. SFBT = Solution-Focused Brief Therapy, D = day of the SFBT (1, 2, 3, 4), R = retest during a given SFBT session (before SFBT, after SFBT), D × R = interaction between a day of SFBT and retest during the SFBT day. *N* = 10.

## Data Availability

The data presented in this study are openly available in Mendeley Data at 10.17632/y5rrgnwtv8.1 reference number [78].

## Supplementary Materials

The following supporting information can be downloaded at: https://www.mdpi.com/article/10.3390/healthcare13162001/s1, entitled: Validation of the Fear of Exams Scale in the sample of university students; including Figure S1: Spearman’s heatmap with correlations between particular items of the Fear of Exam Scale (FoEx-7); Figure S2: The scree plot for the seven-item Fear of Exam Scale (FoEx-7); Figure S3: Path diagram for the seven-item Fear of Exams Scale (FoEx-7); Table S1: Descriptive statistics (*N* = 86); Table S2: Factor loadings (structure matrix); Table S3: Frequentist individual item reliability statistics; questionnaire: “Fear of Exams Scale” in Polish and English language versions. Refs. [83,84,85,86,87,88,89] are cited in supplementary materials.

## Abbreviations

The following abbreviations are used in this manuscript:

STBF	Solution-Focused Brief Therapy
ANOVA	Analysis of variance
RCT	Randomized controlled trial
EARS	Elicit, Acknowledge, Reinforce, Summarize
COVID-19	Coronavirus disease 2019
VAS	Visual Analogue Scale
SPANE	Scale of Positive and Negative Experience
PHQ	Patient Health Questionnaire
GAD	General Anxiety Disorder scale
AIS	Athens Insomnia Scale
