# Supplementary material for: Assessing the Effectiveness of a Solution-Focused Brief Therapy-Based Intervention on Exam Anxiety in First-Year Polish University Students: A Pilot Study with a Randomized Controlled Trial
Source: Healthcare (Basel). 2025 Aug 14;13(16):2001. doi: 10.3390/healthcare13162001 (PMC12385326; doi:10.3390/healthcare13162001)
Supplement: Supplementary file 1 [file healthcare-13-02001-s001.zip › healthcare-3748600-supplementary.pdf]

SUPPLEMENTARY MATERIALS

Validation of the Fear of Exams Scale in the sample of university students

Natalia Cavour-Więcławek, Aleksandra Różańska, & Aleksandra M. Rogowska

Institute of Psychology, University of Opole, Poland

1. Introduction

The 7-item Fear of Exams Scale is derived from the Fear of COVID-19 Scale (Ahorsu et al., 2020). The original scale was created to evaluate participants' agreement with seven statements (e.g., "I cannot sleep because I am worried about getting coronavirus-19"). The initial assessment of the FCV-19S's reliability and validity was performed in Persian, with Ahorsu et al. (2020) noting excellent internal consistency ($\alpha = .82$) and concurrent validity, as the scale showed a positive correlation ($r = .425$) with a measure of anxiety and depression. The FCV-19S has been translated and validated in numerous languages, including English (Winter et al., 2020), and Polish (Chodkiewicz & Gola, 2021). One-factor solution and high reliability was confirmed in previous studies.

The Fear of COVID-19 Scale was adapted for the purpose of assessing exam anxiety among Polish university students, based on previous Polish version of the tool (Chodkiewicz & Gola, 2021) . The modification involved substituting specific terms in the original text: "COVID-19" was changed to "exams," "death from coronavirus" to "consequences of failing exams," "worrying about getting coronavirus" to "worrying about upcoming exams," and "watching news about COVID-19" to "hearing news or discussions about exams." The scale consists of seven items and is evaluated on a 5-point Likert scale, ranging from "I disagree" to "I strongly agree." A higher score on this scale signifies a greater level of exam anxiety. The present study aims to validate the Fear of Exams Scale among Polish university students. One factor structure and high reliability

will be examined, and also criterion validity will be tested using correlations with generalized anxiety disorder symptoms.

2. Materials and methods

2.1. Study design and procedure

The cross-sectional snowball online study was conducted from June 9 to June 17, to investigate the structure and parametric characteristics of the seven-item Fear of Exam Scale (FoEx-7). To decide on the sample size for the Exploratory Factor Analysis (EFA), we followed a common guideline suggesting that the number of participants should be 5 to 10 times the number of items in the scale (de Winter et al., 2009). Given that the FoEx-7 comprises seven items, a sample size of 35 to 70 participants was anticipated. According to a simulation study by de Winter et al. (2009), when data are well-conditioned (e.g., high λ, low f, high p-value), EFA can produce reliable outcomes even with a sample size significantly below 50. We recruited 86 students using online Google Forms questionnaire, which shows adequate sample size. The invitation to the study was performed using e-mailing list by one of the authors of the manuscript (A.R.), who is also academic teacher. The study was performed during the classes and took 10 minutes. All students at university were asked to sent a few invitations to their studying friends.

2.2. Participants characteristics

Mean age of participants was 22 years (ranging from 18 and 31 years; $M = 22.45$, $SD = 2.33$), and prevailed women in the sample ($n = 71$, 83% of the total sample). Most of the respondents were full-time students ($n = 74$, 86% of the total sample), and they studied at first year of the study ($n = 22$, 26%), second year ($n = 9$, 11%), third year ($n = 24$, 28%), fourth year ($n = 27$, 31%), and fifth year ($n = 4$, 5%). Half of students represented psychology faculty ($n = 46$, 54%) and other individuals studied Automation and Robotics, Homeland Security, Medical Biotechnology, Journalism, Electrical Engineering, Pharmaceuticals, Polish Philology, Cultural Studies, Medicine, Pedagogy, Nursing, Social work, and Law.

2.3. Measures

Participants completed a few demographic questions (age, gender, current year and field of study), 7-item Fear of Exam Scale and 7-item General Anxiety Scale (GAD-7). Exam anxiety was assessed using a modified Polish version of the Fear of COVID-19 Scale. The adaptation involved substituting specific terms in the original: "COVID-19" was changed to "exams," "death from coronavirus" to "consequences of failing exams," "worrying about getting coronavirus" to

"worrying about upcoming exams," and "watching news about COVID-19" to "hearing news or discussions about exams." The scale consists of seven items and is rated on a 5-point Likert scale from "I disagree" to "I strongly agree." A higher score on the scale, ranging between 7 and 35, signifies a greater level of exam anxiety.

The Generalized Anxiety Disorder 7-item (GAD-7) scale is a brief self-report questionnaire used to assess the severity of anxiety symptoms. It consists of seven questions, each rated on a 4-point Likert scale, covering aspects of nervousness, worry, restlessness, and fear. The GAD-7 is a widely used tool for screening and monitoring anxiety in both clinical and research settings. Each item is rated on a scale ranging from 0 (not at all) to 3 (nearly every day), allowing for a nuanced assessment of symptom severity. Scores range from 0 to 21, with higher scores indicating more severe anxiety symptoms. he GAD-7 has demonstrated good reliability and validity in various populations, including adolescents and adults. It has shown good internal consistency, with Cronbach's alpha values typically above 0.80. The GAD-7 also exhibits one-factor structure, good criterion and construct validity, correlating well with other measures of anxiety and related conditions.

2.4. Statistical analyses

Descriptive statistics were assessed for FoEx-7 and GAD-7 scales, including minimum (Min.) and maximum value (Max.), median (*Mdn*.), mean (*M*), standard deviation (*SD*), skewness (Skew.), kurtosis (Kurt.), Shapiro-Wilk test (S-W test), and *p*-value for S-W test. The Shapiro-Wilk test was used for assessing univariate normality in the data ($p < 0.05$ indicates a significant departure from normality, leading to rejection of the null hypothesis of normality). Spearman's rank-order correlation coefficient was used to examine correlations between particular items in the FoEx-7 (Dziuban & Shirkey, 1974), and also between fear of exams and generalized anxiety symptoms, to assess criterion validity of the FoEx-7. Preliminary statistical tests were also conducted to verify the assumptions necessary for EFA, which included the Kaiser-Meyer-Olkin Test (KMO) and Bartlett's Test (Bandalos & Finney, 2018). The KMO test evaluates sampling adequacy, determining if the partial correlations among variables are sufficiently low to justify proceeding with factor analysis. KMO values closer to 1 suggest better suitability: KMO > 0.90 excellent; 0.80 - 0.89 good; 0.70 - 0.79 mediocre, 0.60 - 0.69 acceptable, 0.50 - 0.59 marginal, KMO < 0.50 unacceptable for factor analysis. Bartlett's test examines whether the correlation

matrix significantly deviates from an identity matrix, indicating the data's suitability for factor analysis ($p < 0.05$ indicates that the variables are significantly correlated).

The subsequent phase involved conducting an Exploratory Factor Analysis (EFA) (Osborne et al., 2008). To determine the appropriate number of factors to extract, a parallel analysis utilizing principal components was conducted (Dinno, 2014; Hayton et al., 2004). The scree plot was employed to ascertain the number of factors to be included in the model. This plot provides insights into the variance explained by each factor, as indicated by the eigenvalue. For EFA involving non-normal data, the principal axis factoring (PAF) method is recommended, as it does not assume a specific data distribution and is capable of handling non-normal distributions, including those exhibiting skewness or kurtosis. Consequently, the PAF method was utilized to extract factors with an unrotated solution. Reliability was performed using Cronbach's α and McDonald's ω coefficients.

3. Results

3.1. Preliminary statistical analyses

Descriptive statistics are detailed in Table S1. The distribution of variables deviated from normality; consequently, nonparametric tests were employed in subsequent analyses. Spearman's correlation coefficients indicated that all items of the FoEx-7 exhibit moderate to strong intercorrelations (Figure S1), with p-values exceeding 0.001. The KMO value was excellent (KMO = 0.879), suggesting that the correlations among variables are robust enough for factor analysis. Bartlett's test results were $\chi^2(21) = 328.037$, $p < 0.001$, demonstrating that the correlation matrix significantly differs from an identity matrix, implying that the variables are adequately correlated to continue with factor analysis.

Table S1. Descriptive statistics ($N = 86$)

Variable	Min.	Max.	*Mdn.*	*M*	*SD*	Skew.	Kurt.	S-W test	rho
Fear of Exams	7	35	23	22.35	6.72	-0.39	-0.72	0.96*	
Anxiety symptoms	0	21	9.5	10.27	6.60	0.06	-1.31	0.94***	0.79***

Note. *$p < 0.05$, ***$p < 0.001$.

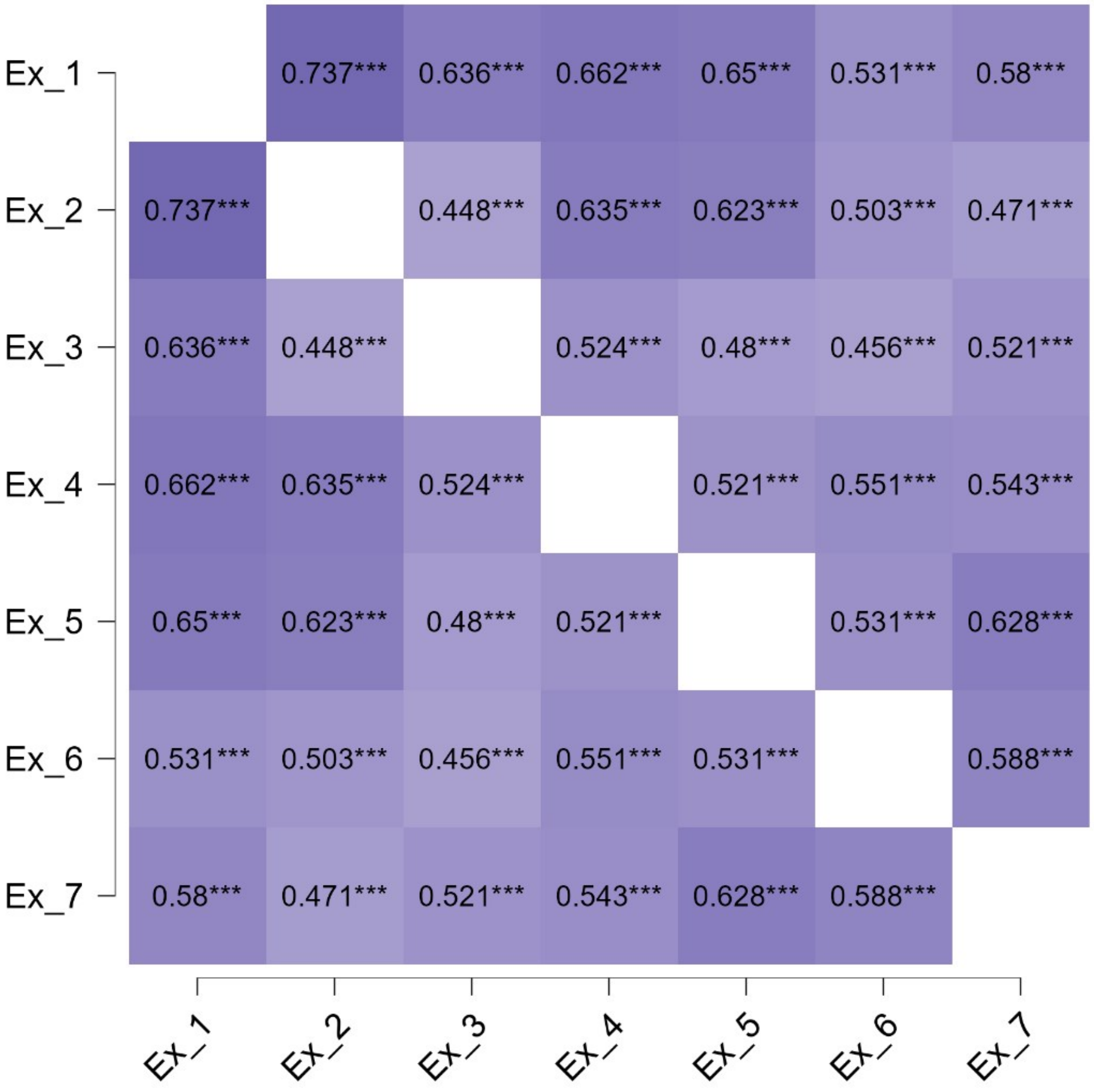

Figure S1. Spearman's heatmap with correlations between particular items of the Fear of Exam Scale (FoEx-7).

Note. Ex_1 = item 1 of the FoEx-7, EX_2 = item 2 of the FoEx-7, EX_3 = item 3 of the FoEx-7, EX_4 = item 4 of the FoEx-7, EX_5 = item 5 of the FoEx-7, EX_6 = item 6 of the FoEx-7, EX_7 = item 7 of the FoEx-7. ***$p < 0.001$.

3.2. Construct validity

As depicted in Figure S2, a one-factor solution was identified, explaining for 56% of fear of exam variance (eigenvalues = 4.37, with 0.56 proportion of variance explained). A minimum

residual factoring method with no rotation method was applied, $\chi^2(14) = 28.56$, $p < 0.011$. Factor loadings for individual items in the model are presented in Table 2 and Figure S3.

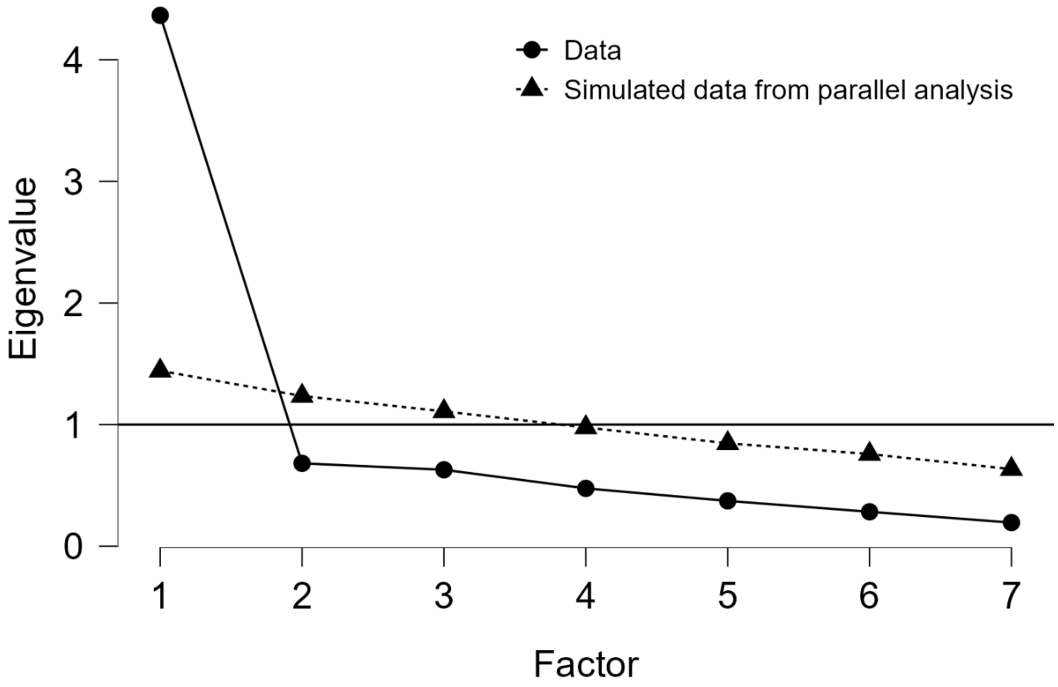

Figure S2. The scree plot for the seven-item Fear of Exam Scale (FoEx-7).

Note. all potential factors represented on the x-axis and the eigenvalues, which indicate the variance explained by each factor, displayed on the y-axis. The horizontal line at an eigenvalue of 1 denotes the Kaiser criterion. The dotted line illustrates the empirical data, while the triangular line represents the simulated data, indicating the parallel analysis. Factors from the empirical data that appear above the simulated data line are included in the model according to the parallel analysis.

Table S2. Factor loadings (structure matrix)

Items	*M*	*SD*	*Mdn.*	Skewness	Kurtosis	Factor loading	Uniqueness
Item 1	3.55	1.11	4	-0.54	-0.27	0.86	0.25
Item 2	4.00	1.16	4	-1.21	0.72	0.81	0.35
Item 3	2.37	1.26	2	0.72	-0.42	0.64	0.59
Item 4	4.07	1.13	4	-1.16	0.48	0.78	0.39
Item 5	3.38	1.23	4	-0.58	-0.68	0.77	0.41
Item 6	2.59	1.31	2	0.41	-0.99	0.67	0.55
Item 7	2.38	1.37	2	0.49	-1.18	0.70	0.51

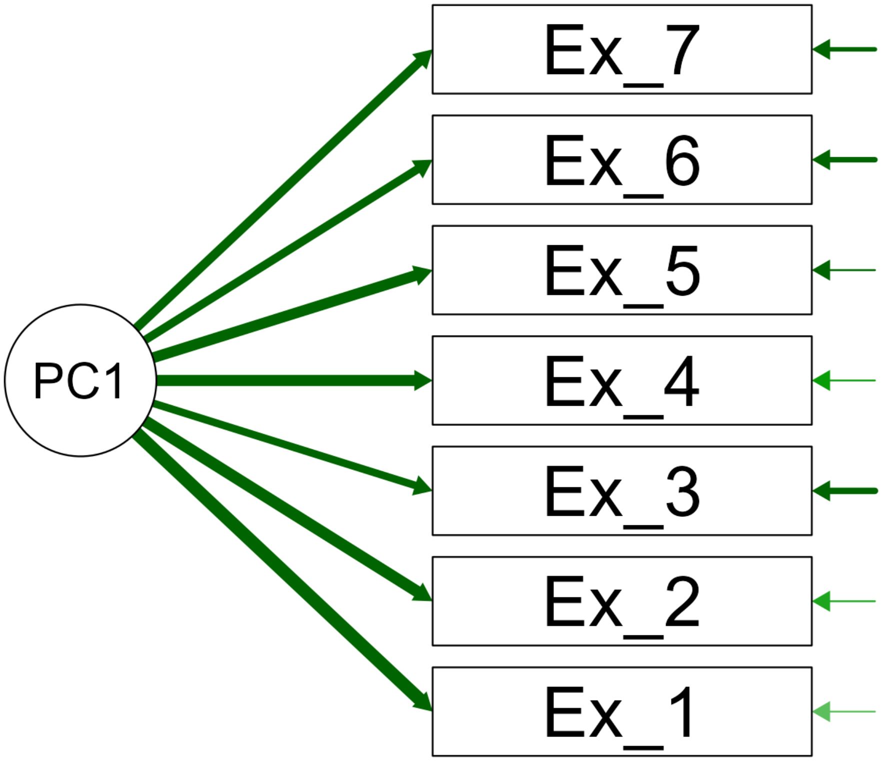

Figure S3. Path diagram for the seven-item Fear of Exams Scale (FoEx-7).

Note. Factors in the model are represented by circles, variables are represented by boxes, and arrows moving from factors to variables represent the loading from the factor to the variable (green indicates a positive loading). The wider the arrows, the stronger the loading. PC1 = principal component 1, Ex_1 = item 1 of the FoEx-7, EX_2 = item 2 of the FoEx-7, EX_3 = item 3 of the FoEx-7, EX_4 = item 4 of the FoEx-7, EX_5 = item 5 of the FoEx-7, EX_6 = item 6 of the FoEx-7, EX_7 = item 7 of the FoEx-7.

3.3. Reliability

The reliability of the FoEx-7 was found to be satisfactory, with Cronbach's α = 0.895 and McDonald's ω = 0.893. An analysis of the reliability of each item within the FoEx-7, when individually removed, indicated that no item warranted exclusion from the questionnaire (Table S3). The average inter-item correlation was 0.56, while the average item-rest correlation was 0.70, with a range from 0.60 to 0.80 (Table S3).

Table S3. Frequentist individual item reliability statistics

Items	ω if item dropped	95% CI		α if item dropped	95% CI		Item-rest correlations
		LL	UL		LL	UL	
Item 1	0.87	0.83	0.91	0.87	0.83	0.91	0.80
Item 2	0.88	0.84	0.92	0.88	0.84	0.91	0.74
Item 3	0.89	0.85	0.93	0.89	0.86	0.92	0.60
Item 4	0.87	0.83	0.92	0.88	0.84	0.91	0.73
Item 5	0.87	0.83	0.92	0.88	0.84	0.91	0.72
Item 6	0.89	0.85	0.92	0.89	0.85	0.92	0.64
Item 7	0.88	0.85	0.92	0.88	0.85	0.92	0.67

Note. ω = McDonald's reliability coefficient, α = Cronbach's reliability coefficient, CI – confidence interval, LL = lower level, UL – upper level.

3.4. Criterion validity

Criterion validity was assessed using Spearman's rho correlation between fear of exams (FoEx-7) and generalized anxiety disorder symptoms (GAD-7), which was determined to be 0.79, $p < 0.001$ (Table S1). Correlation coefficients within the range of 0.7 to 0.9 are generally regarded as high, indicating a strong linear relationship between the two variables, suggesting they tend to vary together. A high correlation coefficient implies that a substantial portion of the variability in fear of exams can be accounted for by its association with the generalized anxiety disorder symptoms.

4. Discussion and conclusions

The study confirmed that the FoEx-7 exhibits a unidimensional structure and demonstrates good reliability, as well as a strong association with generalized anxiety symptoms among Polish university students. Consequently, the scale holds potential for widespread application in future research, particularly within Polish academic settings. Nonetheless, further research is warranted, involving larger sample sizes and a more diverse student population, encompassing various types of universities, different faculties, and improved gender balance. Additionally, cross-cultural studies in an international context could elucidate whether the current structure and parametric properties are influenced by the country of origin.

References

Ahorsu, D.K., Lin, C.Y., Imani, V., Saffari, M., Griffiths, M.D., & Pakpour, A.H. (2020). The Fear of COVID-19 Scale: Development and Initial Validation. *International Journal of Mental Health and Addiction, 20*, 1537–1545. doi:10.1007/S11469-020-00270-8.

Chodkiewicz, J., & Gola, M. (2021). Fear of COVID-19 and Death Anxiety: Polish Adaptations of Scales. *Advances in Psychiatry and Neurology, 30*, 61–72, doi:10.5114/PPN.2021.108471.

de Winter, J. C., Dodou, D., & Wieringa, P. A. (2009). Exploratory Factor Analysis With Small Sample Sizes. *Multivariate behavioral research*, *44*(2), 147–181. https://doi.org/10.1080/00273170902794206

Bandalos, D. L., & Finney, S. J. (2018). Factor analysis: Exploratory and confirmatory. In G. R. Hancock, L. M. Stapleton, & R. O. Mueller, *The reviewer's guide to quantitative methods in the social sciences* (pp. 98-122). Routledge. https://doi.org/10.4324/9781315755649

Dinno, A. (2014) Gently clarifying the application of Horn's parallel analysis to principal component analysis versus factor analysis. *Working paper*. https://alexisdinno.com/Software/files/PA_for_PCA_vs_FA.pdf

Dziuban, C. D., & Shirkey, E. C. (1974). When is a correlation matrix appropriate for factor analysis? Some decision rules. *Psychological Bulletin, 81*(6), 358–361. https://doi.org/10.1037/h0036316

Hayton, J. C., Allen, D. G., & Scarpello, V. (2004). Factor retention decisions in exploratory factor analysis: A tutorial on parallel analysis. *Organizational Research Methods, 7*(2), 191-205. https://doi.org/10.1177/1094428104263675

Osborne, J. W., Costello, A. B., & Kellow, J. T. (2008). Best practices in exploratory factor analysis. In J. Osborne (Ed.), *Best practices in quantitative methods* (pp. 86-99). SAGE Publications, Inc. https://doi.org/10.4135/9781412995627.d8

Winter, T., Riordan, B. C., Pakpour, A. H., Griffiths, M. D., Mason, A., Poulgrain, J. W., & Scarf, D. (2023). Evaluation of the English Version of the Fear of COVID-19 Scale and Its Relationship with Behavior Change and Political Beliefs. *International Journal of Mental Health and Addiction*, *21*(1), 372–382. https://doi.org/10.1007/s11469-020-00342-9

SKALA LĘKU PRZED EGZAMINAMI[1]

(adaptacja: Cavour- Więcławek i in., 2025)

Instrukcja: Oceń, na ile zgadzasz się z poniższymi stwierdzeniami, używając pięciostopniowej skali:

1 – zdecydowanie się nie zgadzam

2 – nie zgadzam się

3 – ani się zgadzam, ani się nie zgadzam

4 – zgadzam się

5 – zdecydowanie się zgadzam

1.	Boję się sesji egzaminacyjnej.	1	2	3	4	5
2.	Czuję dyskomfort, gdy myślę o sesji egzaminacyjnej.	1	2	3	4	5
3.	Pocą mi się dłonie, gdy myślę o egzaminach.	1	2	3	4	5
4.	Boję się niezdania egzaminów i ich konsekwencji.	1	2	3	4	5
5.	Kiedy słyszę wiadomości lub rozmowy o egzaminach, staję się nerwowy/a i niespokojny/a.	1	2	3	4	5
6.	Nie mogę spać, ponieważ martwię się nadchodzącymi egzaminami.	1	2	3	4	5
7.	Dostaję palpitacji serca, gdy myślę o egzaminach.	1	2	3	4	5

[1] Skala Lęku przed Egzaminami (FoEx-7) jest zmodyfikowaną Skalą Lęku przed COVID-19 (Ahorsu i in., 2020; polska adaptacja: Chodkiewicz and Gola, 2021).

Ahorsu, D.K., Lin, C.Y., Imani, V., Saffari, M., Griffiths, M.D., & Pakpour, A.H. (2020). The Fear of COVID-19 Scale: Development and Initial Validation. *International Journal of Mental Health and Addiction, 20*, 1537–1545. doi:10.1007/S11469-020-00270-8.

Chodkiewicz, J., & Gola, M. (2021). Fear of COVID-19 and Death Anxiety: Polish Adaptations of Scales. *Advances in Psychiatry and Neurology, 30*, 61–72, doi:10.5114/PPN.2021.108471.

FEAR OF EXAMS SCALE[2]

(adapted by Cavour- Więcławek i in., 2025)

Instructions: Please indicate to what extent you agree with the following statements, using a five-point scale:

1 – Strongly disagree

2 – Disagree

3 – Neither agree nor disagree

4 – Agree

5 – Strongly agree

1.	I am afraid of exams.	1	2	3	4	5
2.	It makes me uncomfortable to think about exams.	1	2	3	4	5
3.	My hands become clammy when I think about exams.	1	2	3	4	5
4.	I am afraid of failing my exams and the consequences that may follow.	1	2	3	4	5
5.	I feel nervous and anxious when I hear news or people talking about exams.	1	2	3	4	5
6.	I cannot sleep because I worry about exams.	1	2	3	4	5
7.	My heart races or palpitates when I think about exams.	1	2	3	4	5

[2] The 7-item Fear of Exams Scale (FoEx-7) is a modified Fear of COVID-19 Scale (Ahorsu et al., 2020).
Ahorsu, D.K., Lin, C.Y., Imani, V., Saffari, M., Griffiths, M.D., & Pakpour, A.H. (2020). The Fear of COVID-19 Scale: Development and Initial Validation. *International Journal of Mental Health and Addiction, 20*, 1537–1545. doi:10.1007/S11469-020-00270-8.
